# Phase 3 randomized, double-blind, sham-controlled Trial of e-TNS for the Acute treatment of Migraine (TEAM)

**DOI:** 10.1038/s41598-022-09071-6

**Published:** 2022-03-24

**Authors:** Deena E. Kuruvilla, Joseph I. Mann, Stewart J. Tepper, Amaal J. Starling, Gregory Panza, Michael A. L. Johnson

**Affiliations:** 1 Department of Neurology, Westport Headache Institute, Westport, CT USA; 2 Department of Neurology, Rochester Clinical Research, Rochester, NY USA; 3 Department of Neurology, Dartmouth-Hitchcock Medical Center, Lebanon, NH USA; 4 Department of Neurology, Mayo Clinic, Scottsdale, AZ USA; 5 Department of Research, Hartford Healthcare, Hartford, CT USA; 6 Department of Medical Affairs, CEFALY Technology, 4102 Seraing, Belgium

**Keywords:** Neurological disorders, Headache, Migraine, Health care, Therapeutics, Pain management

## Abstract

Migraine is one of the most common and debilitating neurological disorders worldwide. External Trigeminal Nerve Stimulation (e-TNS) is a non-pharmacological, non-invasive therapeutic alternative for patients with migraine. The TEAM study was a prospective, multicenter, randomized, double-blind, sham-controlled, Phase 3 trial for 2-h, continuous, e-TNS treatment of a single moderate or severe migraine attack at home. A total of 538 adults meeting the International Classification of Headache Disorders 3rd edition criteria for 2–8 migraine headache days per month were recruited and randomized in a 1:1 ratio to 2-h active or sham stimulation. Migraine pain levels and most bothersome migraine-associated symptoms (MBS) were recorded at baseline, 2 h, and 24 h using a paper diary. The primary endpoints for the study were pain freedom at 2 h and freedom from the MBS at 2 h. The secondary endpoints were pain relief at 2 h, absence of most bothersome migraine-associated symptoms (MBSs) at 2 h, acute medication use within 24 h after treatment, sustained pain freedom at 24 h, and sustained pain relief at 24 h. Adverse event data was also collected and compared between groups. Five hundred thirty-eight patients were randomized to either the verum (*n* = 259) or sham (*n* = 279) group and were included in an intention-to-treat analysis. The percentage of patients with pain freedom at 2 h was 7.2% higher in verum (25.5%) compared to sham (18.3%; p = 0.043). Resolution of most bothersome migraine-associated symptom was 14.1% higher in verum (56.4%) compared to sham (42.3%; p = 0.001). With regards to secondary outcomes, pain relief at 2 h was 14.3% higher in verum (69.5%) than sham (55.2%; p = 0.001), absence of all migraine-associated symptoms at 2 h was 8.4% higher in verum (42.5%) than sham (34.1%; p = 0.044), sustained pain freedom and pain relief at 24 h was 7.0% and 11.5% higher in verum (22.8 and 45.9%) than sham (15.8 and 34.4%; p = 0.039 and .006, respectively). No serious adverse events were reported. Treatment with 2-h e-TNS is a safe and effective, non-invasive, and non-pharmacological alternative for the acute treatment of migraine attacks in an at-home setting.

**Trial registration** Clinicaltrials.gov Identifier: NCT03465904. Registered 14/03/2018. https://www.clinicaltrials.gov/ct2/show/record/NCT03465904.

## Introduction

Migraine is one of the most common neurological disorders and has been ranked by the World Health Organization as the world's second leading cause of disability^1^.

Currently, the first line acute treatments available for migraine include triptans, non-steroidal anti-inflammatory medications (NSAIDS), lasmiditan, and gepants (ubrogepant and rimegepant)^2–5^. Acute pharmacological treatments for migraine may result in intolerable side effects, may be contraindicated in patients with cardiovascular and/or cerebrovascular disease, or may lack efficacy for pain freedom^6,7^. Consequently, this leaves up to 40% of episodic migraine patients with unmet migraine treatment needs^8^. Another concern in the acute treatment of migraine is acute medication overuse. The frequent intake of acute migraine medications may lead to an increased risk of adverse events, migraine chronification, and medication overuse headache (MOH)^9–12^.

Studies have shown that a majority of patients use non-pharmacological approaches for migraine treatment^13,14^. External Trigeminal nerve stimulation (e-TNS) is a non-pharmacological, non-invasive therapeutic alternative for patients with migraine who do not respond to pharmacologic acute migraine therapies, have intolerances/contraindications to acute migraine therapies, or prefer to avoid pharmaceutical therapies. A few proposed mechanisms exist for the role of e-TNS treatment for migraine. The role of the trigeminovascular system in migraine has been well established. The supraorbital nerve which is stimulated directly by e-TNS, is a branch of the first division of the trigeminal nerve. FDG-PET studies have shown that e-TNS may modulate the function of pain-controlling brain regions^15^.

Several trials, including the sham-controlled Acute Migraine therapy with External Trigeminal Neurostimulation (ACME) clinical trial, demonstrated efficacy and safety of e-TNS in the acute treatment of migraine attacks^16–19^. E-TNS treatment in the ACME trial took place in a hospital setting, with a limited sample size and after a migraine duration of 4 h prior to intervention. Additionally, the findings of the ACME trial indicated a trend of longer treatment durations favoring improved migraine relief. However, to the best of our knowledge, no data are available from a clinical trial using similar protocol design and endpoints in a real-world, at-home setting. A single-center open-label trial of a patient's self-administering 2-h e-TNS treatments for acute treatment of a migraine attack suggested favorable outcomes in 2-h pain freedom, most bothersome migraine-associated symptom, and sustained pain freedom at 24-h^20^.

The objective of the Trial of e-TNS for the Acute treatment of Migraine (TEAM) study is to evaluate the efficacy and safety of self-administered, 2-h (two consecutive 1-h) e-TNS treatments for migraine attacks, with and without aura, in an at-home setting.

## Methods

### Standard protocol approvals and patient consents

The study protocol and informed consent form were reviewed and approved by the ADVARRA (formerly IntegReview) Investigational Review Board, protocol #51401, on February 15, 2018 and approved at all sites. The study was also registered with Clinicaltrials.gov Identifier: NCT03465904, 14/03/2018. Written informed consent was obtained from all participants. The full protocol design is included with Supplementary Materials.

### Study design and participants

This study was a prospective, multi-center, randomized, double-blind, sham-controlled study conducted across ten centers in the United States (Rochester Clinical Research in Rochester NY, Coastal Carolina Research Center in Charleston, SC, Rapid Medical Research Inc. in Cleveland OH, Palm Beach Research Center in West Palm Beach, FL, Clinical Research Consortium in Las Vegas, NV, Clinical Research Consortium in Phoenix, AZ, Pharmacology Research Institute in Encino, CA, Yale University New Haven, CT, Meridian Clinical Research in Savannah, GA, and Meridian Clinical Research Rockville, MD.). This trial used the traditional protocol design and endpoints for many acute migraine medications, following FDA guidance and International Headache Society guidelines^21–24^.

Men and women adults (aged 18–65 years) were eligible for enrollment if (1) they had a diagnosis of episodic migraine with or without aura, for at least 1 year, according to the diagnostic criteria listed an International Classification of Headache Disorders, Third edition (ICHD-3)^12^, (2) migraine diagnosis before 50 years of age, and (3) 2 to 8 monthly migraine frequency with moderate to severe intensity. The inclusion and exclusion criteria are listed in Table 1.

**Table 1:** Inclusion and exclusion criteria.

**Inclusion criteria**
1. Age from 18 to 65 on the day of signing the informed consent form
2. ≥ 1-year history of migraine with or without aura according to the diagnostic criteria listed in International Classification of Headache Disorders (ICHD) III beta (2013) section 1, migraine (10), with the exception of aura without headache, hemiplegic migraine, and brainstem aura migraine
3. Migraine onset before the age of 50
4. Having between 2 and 8 moderate or severe migraine attacks (Grade 2 or 3) per month in each of the 2 months prior to screening
5. Patient understands the study procedures, alternative treatments available, and voluntarily agrees to participate in the study by giving written informed consent
6. Patient is able to read and understand the written information (instruction sheet, paper diary and AE reporting form)
**Exclusion criteria**
1. Patient has difficulty distinguishing his/her migraine attacks from tension-type headaches
2. Patient has more than 15 headache days per month
3. Patient having received supraorbital nerve blocks in the prior 4 months
4. Patient having received Botox treatment in the prior 4 months
5. Modification of a migraine prophylaxis treatment in the previous 3 months
6. Diagnosis of other primary headache disorders, except rare (< 4) tension-type headaches per month
7. Diagnosis of secondary headache disorders including Medication Overuse Headache
8. Patient abusing opioids or use of recreational or illicit drugs or having had a recent history (within the last year) of drug or alcohol abuse or dependence
9. Implanted metallic or electronic device in the head
10. Cardiac pacemaker or implanted or wearable defibrillator
11. Patient having/had previous experience with the Cefaly^®^ device
12. Migraine Aura without a headache
13. Patient is currently participating or has participated in a study with an investigational compound or device in the last 30 days before the screening visit
14. Patient not having the ability to appropriately use the device and/or to perform himself/herself or bear the first 20-min stimulation session during the training test session at the study site

*ICHD-III* International Classification of Headache Disorders, Third edition.

After enrollment and prior to randomization, patients were trained to use the migraine diary, the application and operation of the e-TNS device, and then were tested with a 20-min nociceptive assessment. Patients were eligible for randomization if they met all inclusion criteria, none of the exclusion criteria, and demonstrated capability to self-apply, operate, and tolerate the device stimulation.

### Neurostimulation device

Neurostimulation was applied with an e-TNS device (CEFALY^®^ Technology, Seraing, Belgium) for a 2-h, continuous session. The device is a constant current generator for a maximum skin impedance of 2.2 kΩ that delivers rectangular biphasic symmetrical pulses with a zero electrical mean^25^. Verum and sham devices used identical rectangular biphasic symmetrical pulses of 250 μs, with a width that induced paresthesia. The sham device provided low frequency pulses of 3 Hz, while the verum device produced high frequency pulses of 100 Hz. During stimulation, the intensity increases linearly to reach a maximum of 16 mA after 14 min. After 14 min, the stimulation intensity remains constant for the remainder of the treatment (106 min). The total dose of current delivered during a 2-h session is 2.728 coulombs^25,26^.

The electrical pulses are transmitted transcutaneously via a supraorbital bipolar self-adhesive electrode (30 × 94 mm^2^) placed on the forehead and designed to cover and excite (trigger action potentials) the supratrochlear and supraorbital nerves bilaterally.

### Randomization and masking

**Figure 1:**
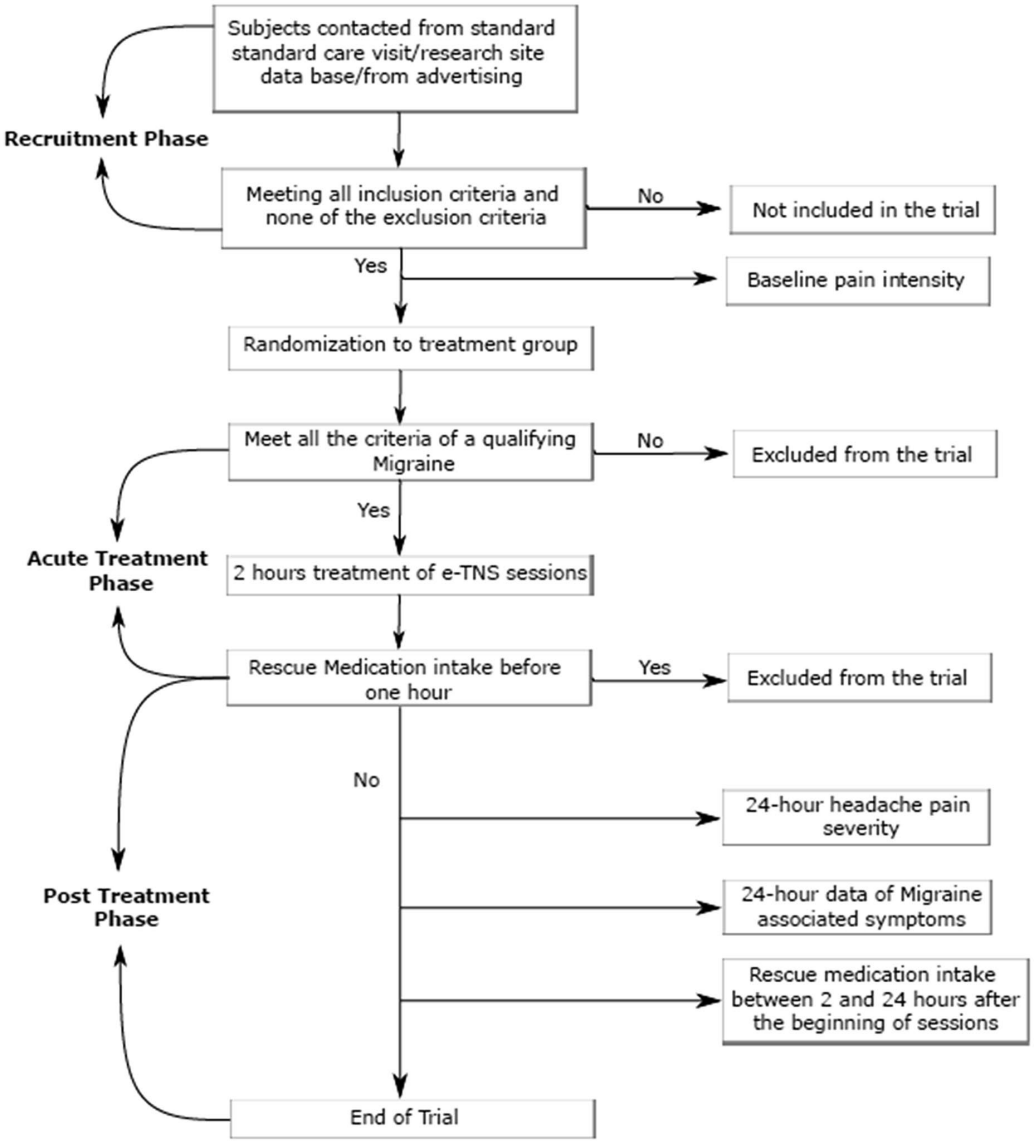
Study design flowchart.

The overall study design flow is illustrated in Fig. 1. Subjects who met all of the study criteria were randomly assigned 1:1 to either the verum or sham group and were provided with a device and a diary to keep with them for 2 months to be used on a qualifying migraine attack. The devices were sequentially numbered following a random allocation sequence generated by the research and development department of the device manufacturer and stratified by center with a 1:1 allocation to treatment groups (verum or sham) using a block size of four. The verum and sham devices were identical in hardware composition and function. The only difference was in the stimulation frequency of the sham device at 3 Hz, below the clinical threshold of sensory detection^25^. Blinding between verum and sham stimulations was validated by a test at Spincontrol Laboratory (Tours, France) in which participants were unable to differentiate between sham and verum stimulation. The study participants and care providers were blinded to intervention assignments. If a subject had not treated a migraine attack within 2 months following the screening visit, the study material (Cefaly^®^ Acute Program device with accessories) was required to be returned to the study site.

### Procedures

Each patient received a migraine diary and device to record and treat one qualifying migraine within a 2-month period. A qualifying migraine attack had to meet the following criteria: (1) a migraine with moderate or severe headache intensity, and (2) with at least one migraine-associated symptom, being photophobia, phonophobia, nausea, and/or vomiting. The patients were instructed to self-administer the e-TNS treatment, according to the training and instruction, within 4 h of migraine onset or within 4 h of awakening with a migraine headache. Migraines occurring within 48 h of a prior migraine did not qualify for e-TNS treatment. Patients were also advised not to start e-TNS treatment for a migraine spontaneously resolving. In addition, patients were advised to not take any acute migraine medications prior to or during e-TNS therapy. Patients were permitted to use acute migraine therapy after a 2-h e-TNS treatment was completed if needed.

During the acute migraine phase, patients reported the following data in their headache diary: (1) pre-treatment (baseline) migraine pain severity (no pain, mild pain, moderate pain severe pain); (2) presence of migraine associated-symptoms (photophobia, phonophobia, nausea, and/or vomiting); (3) which migraine-associated symptom was their most bothersome symptom (MBS); and (4) any adverse effects. Patients were advised to record these data prior to the start of the e-TNS session. The same data were recorded at 2 h (following e-TNS treatment), and at 24 h (post-treatment). Patients were also asked to record intake of any acute anti-migraine medication between 2 and 24-h after the start of e-TNS treatment. Patients recorded all adverse event (AE) dates, nature, treatment (if any), and progress in a provided AE collection form. Patients were instructed to notify the investigator immediately for any serious or severe AE experienced with the stimulation.

At the conclusion of the 2-month period, subjects were required to return their headache diary and e-TNS device. The e-TNS device for each subject was interrogated for proper device functioning, and duration of stimulation delivered. Subjects who failed to return the migraine diary or had the absence of a qualifying migraine were excluded from the analysis.

### Outcomes measures

The primary outcome measures included pain freedom at 2 h and resolution of most bothersome migraine-associated symptom at 2 h from the beginning of e-TNS therapy for one qualifying migraine attack. The secondary outcomes measures included (1) pain relief at 2 h defined as a reduction of a moderate or severe migraine headache at baseline to a mild or no headache, (2) resolution of any migraine-associated symptom at 2 h after beginning of e-TNS treatment, (3) sustained pain freedom, defined as pain freedom at 2 h and pain freedom at 24 h without the use of anti-migraine medication during those 24 h, (4) sustained pain relief at 24 defined as mild or no headache at 2 h and mild or no headache at 24 h after beginning the eTNS session without the use of anti-migraine medication during those 24 h, and (5) use of a rescue medication between 2–24 h after beginning of e-TNS session.

### Statistical methods

Identical statistical analyses were performed for the intent-to-treat (ITT) and the per protocol (PP) populations. The ITT population consisted of all randomized patients, who received any duration of sham or verum treatment and returned completed study materials. The PP population consisted of patients who strictly met inclusion criteria and completed at least 60 min of treatment. Patient exclusion from the PP analysis is displayed in Table 2.

**Table 2:** Patient characteristics.

Variable	ITT analysis				Per protocol analysis			
	Verum (*n* = 259)	Sham (*n* = 279)	Total (*N* = 538)	*P*	Verum (*n* = 207)	Sham (*n* = 231)	Total (*N* = 438)	*P*
Age (yr)	40.22 ± 11.62	42.0 ± 12.30	41.14 ± 12.00	0.087	40.64 ± 11.25	40.85 ± 11.84	40.75 ± 11.56	0.847
**Gender**								
Female	214 (82.6)	229 (82.1)	443 (82.3)	0.868	173 (83.6)	191 (82.7)	364 (83.1)	0.804
Male	45 (17.4)	50 (17.9)	95 (17.7)		34 (16.4)	40 (17.3)	74 (16.9)	
**Headache pain at baseline**								
No pain	1 (0.4)	0 (0.0)	1 (0.2)	0.532	0 (0.0)	0 (0.0)	0 (0.0)	0.552
Mild	1 (0.4)	0 (0.0)	1 (0.2)		1 (0.5)	0 (0.0)	1 (0.2)	
Moderate	155 (59.8)	166 (59.5)	321 (59.7)		125 (60.4)	143 (61.9)	268 (61.2)	
Severe	102 (39.4)	113 (40.5)	215 (40.0)		81 (39.1)	88 (38.1)	169 (38.6)	
**Symptoms at baseline**								
Photophobia	247 (95.4)	259 (92.8)	506 (94.1)	0.214	198 (95.7)	214 (92.6)	412 (94.1)	0.183
Phonophobia	207 (79.9)	228 (81.7)	435 (80.9)	0.529	164 (79.2)	189 (81.8)	353 (80.6)	0.482
Nausea	169 (65.3)	172 (61.6)	341 (63.4)	0.386	139 (67.1)	144 (62.3)	283 (64.6)	0.293
Vomiting	23 (8.9)	24 (8.6)	47 (8.7)	0.909	18 (8.7)	17 (7.4)	35 (8.0)	0.607
**MBS at baseline**								
Photophobia	162 (62.5)	183 (65.6)	345 (64.1)	0.685	133 (64.3)	149 (64.5)	282 (64.4)	0.469
Phonophobia	38 (14.7)	35 (12.5)	73 (13.6)		28 (13.5)	32 (13.9)	60 (13.7)	
Nausea	55 (21.2)	54 (19.4)	109 (20.3)		45 (21.7)	45 (19.5)	90 (20.5)	
Vomiting	4 (1.5)	7 (2.5)	11 (2.0)		1 (0.5)	5 (2.2)	6 (1.4)	
Migraine with aura	113 (43.6)	111 (39.8)	224 (41.6)	0.366	87 (42.0)	88 (38.1)	175 (40.0)	0.401
Duration of treatment	120.0, 30.0	120.0, 24.0	120.0, 25.0	U = 35,011, 0.463	120.0, 0.0	120.0, 1.0	120.0, 0.0	U = 21,014, **0.002**

Categorical data presented as # (%). Normally distributed continuous data presented as mean±SD. Non-normally distributed continuous data presented as median, IQR and a Mann Whitney U test was used to compare groups. *MBS* most-bothersome symptom.

Our power analysis indicated that a minimum of 239 patients per group was needed to provide 90% power to detect a 13.4% difference in the primary outcome, pain freedom at 2 h. Accounting for an estimated attrition rate of 20%, we aimed to enroll 299 patients per group. Data were assessed for normality and to confirm that test assumptions were met. Means and standard deviations (SD) summarized continuous, normally distributed data; continuous, non-normally distributed data are summarized with medians and interquartile ranges (IQR). Categorical data are presented as frequencies and percentages. All analyses were performed using SPSS, version 26.0 with two-sided levels of statistical significance established at *p* < 0.05.

A Chi-square test of independent proportions was used to compare primary and secondary efficacy outcomes between groups. A Fisher’s exact test was used (due to cell counts < 5) to compare the proportion of each type of adverse event between groups. In addition, a multivariate logistic regression analysis using a hierarchical approach was performed for each efficacy outcome as the dependent variable and treatment group as the primary independent variable, while controlling for potential confounders in the model including age, duration of treatment (minutes), and having an adverse effect (yes/no).

### Ethics approval

The study protocol and informed consent form were reviewed and approved by the IntegReview Investigational Review Board on February 15, 2018 protocol #51401, and approved at all sites. The study was conducted in accordance with Good Clinical Practice guidelines, the principles of the Declaration of Helsinki, and all applicable regulatory requirements. All participants of the study provided written informed consent prior to initiation of any study procedures.

## Results

**Figure 2:**
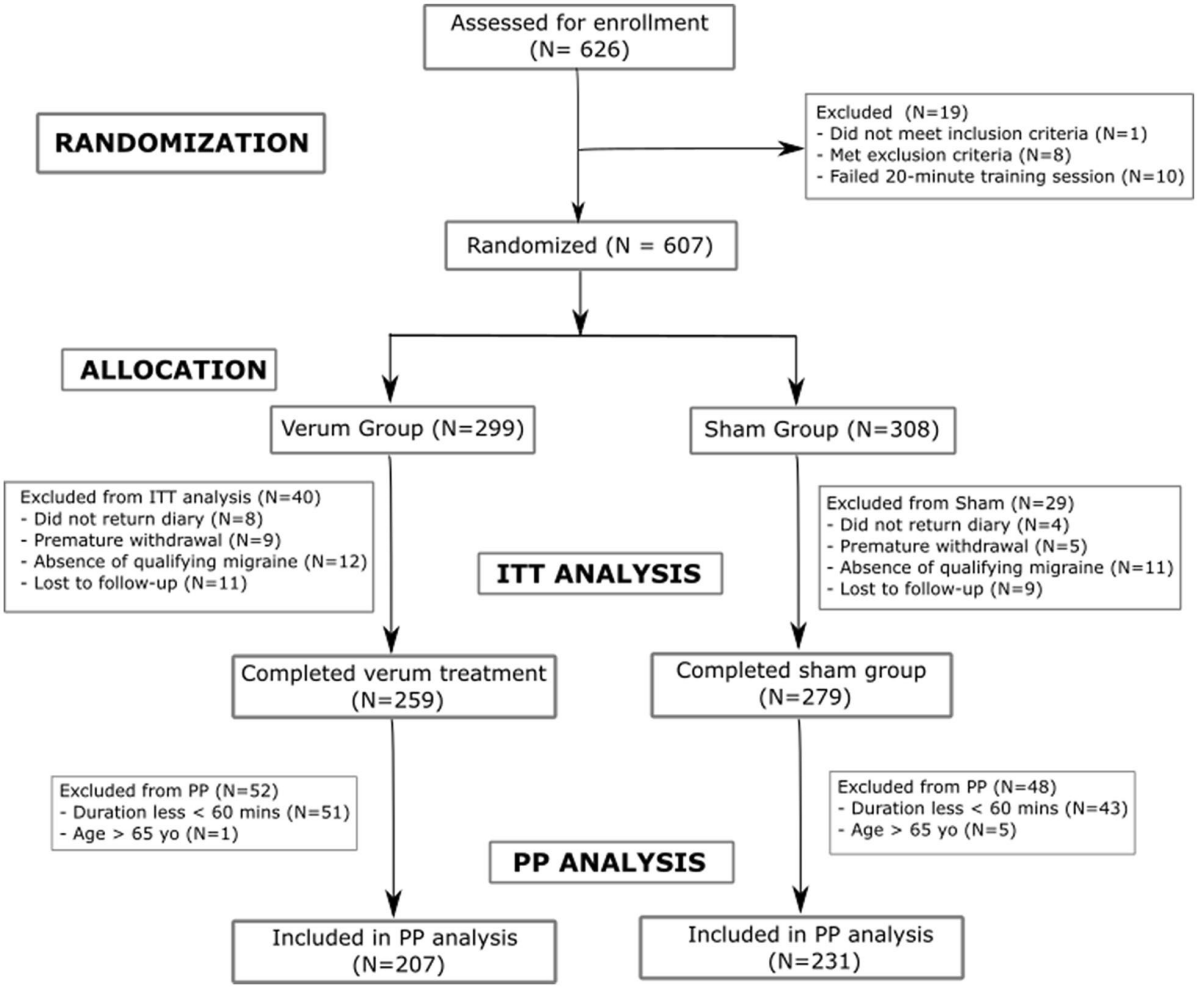
Patient enrollment and allocation.

Between April 10, 2018 and January 11, 2019, 626 subjects were screened for eligibility, and 607 were randomized (601 patients met eligibility requirements, however six were older than age 65 and were randomized in error and included in the ITT analysis). Twenty-three (23) patients had absence of a qualifying migraine, 20 were lost to follow-up, 14 patients withdrew from the study, and 12 did not return their diary and study materials, resulting in a total of 538 patients (verum *n* = 259, sham *n* = 279) which were included in the intention-to-treat (ITT) analysis. Figure 2 summarizes the eligibility, randomization, and inclusion of subjects in the ITT and PP analyses. Patient characteristics are summarized in Table 2.

There were no statistically significant differences in any patient characteristics between groups including age, gender, headache pain severity, headache symptoms at baseline, migraine with and without aura, and duration of treatment (all *p*s > 0.05).

### Outcome measures

For succinctness, ITT analysis results are described in text and presented in results tables, while PP analysis results are only presented in results tables. With regards to primary outcome measures (Table 3), the percentage of patients experiencing pain freedom at 2 h after the beginning of e-TNS was higher in the verum (25.5%) compared to sham groups (18.3%), for a therapeutic gain of 7.2% (*p* < 0.05). The percentage of patients experiencing resolution of most bothersome migraine-associated symptom at 2-h after beginning of e-TNS was similarly higher in the verum (56.4%) compared to sham group (42.3%), for a therapeutic gain of 14.1% (*p* < 0.01).

**Table 3:** Headache pain outcomes following e-TNS session: Verum vs. Sham.

Variable, # (%)	ITT analysis						Per protocol analysis					
	Verum (*n* = 259)	Sham (*n* = 279)	Total (*N* = 538)	*X*^2^-statistic	ES	*P*	Verum (*n* = 207)	Sham (*n* = 231)	Total (*N* = 438)	*X*^2^-statistic	ES	*P*
Pain freedom at 2 h	66 (25.5)	51 (18.3)	117 (21.7)	4.10	0.18	**0.043**	57 (27.5)	44 (19.0)	101 (23.1)	4.434	0.20	**0.035**
Absence of MBS at 2 h	146 (56.4)	118 (42.3)	264 (49.1)	10.65	0.28	**0.001**	119 (57.5)	100 (43.3)	219 (50.0)	8.803	0.28	**0.003**
Pain relief at 2 h	180 (69.5)	154 (55.2)	334 (62.1)	11.67	0.30	**0.001**	151 (72.9)	132 (57.1)	283 (64.6)	11.925	0.35	**0.001**
Absence of all MAS at 2 h	110 (42.5)	95 (34.1)	205 (38.1)	4.04	0.18	**0.044**	95 (45.9)	79 (34.2)	174 (39.7)	6.236	0.24	**0.013**
Rescue meds at 2–24 h	82 (31.7)	105 (37.6)	187 (34.8)	2.11	− 0.12	0.146	67 (32.4)	90 (39.0)	157 (35.8)	2.064	− 0.14	0.151
Pain freedom at 24 h	59 (22.8)	44 (15.8)	103 (19.1)	4.26	0.18	**0.039**	50 (24.2)	38 (16.5)	88 (20.1)	4.036	0.20	**0.045**
Pain relief at 24 h	119 (45.9)	96 (34.4)	215 (40.0)	7.45	0.24	**0.006**	94 (45.4)	84 (36.4)	178 (40.6)	3.704	0.18	0.054

*MBS* most bothersome symptom, *MAS* migraine-associated symptoms, *ES* effect size.

With regards to secondary outcomes (Table 3), pain relief at 2 h was higher in the verum (69.5%) compared to sham groups (55.2%), for a therapeutic gain of 14.3% (*p* < 0.01). Absence of any migraine-associated symptoms at 2 h was also higher in verum (42.5%) compared to sham (34.1%), with a therapeutic gain of 8.4% (*p* < 0.05). Sustained pain freedom (22.8% vs. 15.8, *p* < 0.05) and sustained pain relief at 24 h (45.9% vs. 34.4%, *p* < 0.01) were higher in verum compared to sham, with therapeutic gains of 7% and 11.5%, respectively. There was no statistically significant difference between groups for use of rescue medication between 2 and 24 h post treatment. The categories of anti-migraine rescue medications reported by patients are summarized in Supplemental Table 1.

Multivariate logistic regression analysis indicated that treatment group was a significant predictor of all primary and secondary outcomes, while controlling for potential confounders, with the exception of rescue medication use between 2- and 24-h post-treatment. These results are consistent with the results from the primary and secondary bivariate analysis. The multivariate regression analysis also indicated that in the overall sample, duration of treatment (minutes) was a predictor of pain relief at two hours (*p* < 0.05), having an adverse event was a predictor for using rescue medication (*p* < 0.05), and age was a significant predictor of sustained pain freedom (*p* < 0.05) and sustained pain relief (*p* < 0.01) at 24 h.

### Adverse events and compliance

The percentage of patients that reported an adverse event was higher in verum (8.5%) compared to sham (2.9%, *p* < 0.01). Table 4 summarizes the incidence and types of adverse events in each group. There were no serious adverse events encountered in this study. All adverse events were minor and fully reversible without intervention or additional treatment after cessation of treatment. Treatment-related forehead paresthesia, discomfort, and burning were the only adverse events significantly higher in the verum (3.5%, 9 patients) compared to the sham group (0.4%, 1 patient; *p* < 0.01). All other adverse events did not reach statistical significance between groups (all *p*s > 0.05).

**Table 4:** Adverse events.

Adverse event, # (%)	Verum (*n* = 259)	Sham (*n* = 279)	Total (*N* = 538)	*P*	Verum (*n* = 207)	Sham (*n* = 231)	Total (*N* = 438)	*P*
Forehead paresthesias, discomfort or burning	9 (3.5)	1 (0.4)	10 (1.9)	**0.009**	4 (1.9)	1 (0.4)	5 (1.1)	0.194
Nausea/vomiting	4 (1.5)	0 (0.0)	4 (0.7)	0.053	3 (1.4)	0 (0.0)	3 (0.7)	0.105
Dizziness	1 (0.4)	2 (0.7)	3 (0.6)	1.000	1 (0.5)	1 (0.4)	2 (0.5)	1.000
Neck stiffness/muscle tension	1 (0.4)	2 (0.7)	3 (0.6)	1.000	1 (0.5)	2 (0.9)	3 (0.7)	1.000
Worsened headache	2 (0.8)	0 (0.0)	2 (0.4)	0.231	0 (0.0)	0 (0.0)	0 (0.0)	–
Orodynia—tooth or jaw pain	1 (0.4)	1 (0.4)	2 (0.4)	1.000	0 (0.0)	1 (0.4)	1 (0.2)	1.000
Restlessness	1 (0.4)	1 (0.4)	2 (0.4)	1.000	1 (0.5)	1 (0.4)	2 (0.5)	1.000
Abdominal discomfort or cramping	0 (0.0)	2 (0.7)	2 (0.4)	0.500	0 (0.0)	0 (0.0)	0 (0.0)	–
Dry mouth	1 (0.4)	0 (0.0)	1 (0.2)	0.481	1 (0.5)	0 (0.0)	1 (0.2)	0.473
Excessive sweating	2 (0.8)	0 (0.0)	2 (0.4)	0.231	2 (1.0)	0 (0.0)	2 (0.5)	0.223
Sedation/sleepiness	1 (0.4)	0 (0.0)	1 (0.2)	0.481	1 (0.5)	0 (0.0)	1 (0.2)	0.473
Total patients with an adverse effect	22 (8.5)	8 (2.9)	30 (5.6)	**0.004**	14 (6.8)	5 (2.2)	19 (4.3)	**0.018**

Fisher’s exact test is used for all comparisons due to cell counts < 5, and therefore *X*^2^ statistic is not presented. ‘Total patients with an adverse effect’ does not add up to the total number of adverse effects in separate categories due to one patient in each group having two adverse events.

Total compliance, defined as treatment duration of 120 min and ≥ 60 min, was 65.6% and 82.5% respectively. For both 120 min and ≥ 60 min treatment duration there were no differences in compliance between verum (69.1% and 80.3% respectively) and sham (62.4% and 84.6% respectively) groups.

## Discussion

The prospective, multicenter TEAM trial provides the first Phase 3 evidence demonstrating efficacy and safety of patient-administered, 2-h e-TNS treatment for acute treatment of migraine with and without aura in the out-of-hospital setting. We will discuss the outcomes and safety findings, address practical implications of these findings, and then examine limitations of this study.

Prior studies of e-TNS for acute treatment of migraine attacks suggested longer duration of therapy may result in improved pain freedom and sustained migraine relief^16,17^. In the TEAM study, 2-h e-TNS treatment in acute migraine demonstrated significantly higher rates of migraine pain freedom at 2 h, and resolution of migraine associated MBS compared to sham. In addition, 2-h e-TNS for acute treatment of migraine attacks resulted in an increased rate of pain relief at 2 h, resolution of all migraine-associated symptoms, and sustained pain freedom and sustained pain relief at 24 h when compared to the sham group. These findings substantiate prior hypotheses that duration of e-TNS treatment is a significant parameter in achieving optimal pain relief^12–14,16^.

The percentage of patients experiencing pain freedom after 2-h of e-TNS was lower in the TEAM study than previously reported in a non-sham controlled, open-label Rochester trial (25.5 vs. 35.6%, respectively). However, the TEAM study and the precedent open-label trial^17^ found similar results for the proportion of patients experiencing MBS resolution (56.4% vs. 60.4%), sustained 24-h pain freedom (23.2% vs. 25%), sustained pain relief at 2-h (69.5% vs. 70.8%), and absence of all migraine-associated symptoms (42.5 vs. 48.8%). These findings indicate consistent evidence supporting efficacy of 2-h e-TNS in the management of acute migraine with and without aura.

There were no significant differences in the use of acute migraine medication use at 2 and 24-h between verum and sham groups. This finding was also consistent with the ACME trial. Though e-TNS is shown to be an effective and safe, non-invasive, and non-pharmacological alternative to acute migraine pharmaceuticals, the concordance of these findings may support the use of e-TNS as an augmentative or complementary therapy in the at-home setting. Further study is needed to elucidate the applied role of e-TNS in a stratified-approach to acute treatment of migraine. Although the stimulation frequency between the sham and verum differed in this study, it remains unclear to what extent the sham stimulation provided a partial therapeutic effect as opposed to a true placebo effect^27^. Prior e-TNS studies demonstrating efficacy used similar sham stimulation parameters^18–20^. A future randomized, self-control longitudinal study, which allows subjects to treat more than one migraine attack over a 3–4-month period, would be helpful in assessing the consistency of the response of acute e-TNS treatment in migraine attacks^27^.

As an aggregate, patients with migraine with aura appeared to have a more robust response to e-TNS stimulation in both sham and verum groups. There were no consistent differences in response between verum and sham groups. The mechanism of these findings is unclear. Patients with migraine aura may have early indicators of migraine onset allowing them to identify and treat migraine earlier. Several studies of acute migraine medications demonstrate early migraine detection and treatment is associated with greater likelihood of migraine treatment succes^28–31^. In the TEAM study, the e-TNS start time from migraine onset was not obtained. Further studies are needed to clarify the effectiveness of e-TNS therapy early versus late in the migraine attack trajectory.

There were no serious adverse events and all non-device and device-related adverse events were minor and fully reversible without intervention after cessation of treatment. The most common adverse side effect of forehead paresthesia and discomfort was consistent with findings of prior studies^14,17,32,33^. Unlike prior studies, the proportion of patients reporting this device-related adverse effect was higher in the verum compared to the sham group. Although the actual number that experienced this adverse event was small (3.9%), this finding may indicate that 2-h e-TNS treatment is associated with a higher incidence of forehead paresthesia.

The presence of adverse events and tolerance of e-TNS therapy is important in the outcome of migraine relief. In a prior survey of 2313 patients using e-TNS, the rate of adverse events was higher in patient’s unsatisfied with therapy. In this survey, 2% of respondents stopped e-TNS therapy due to adverse events, and approximately 9% of patients with suboptimal compliance reported adverse events^33^. In the TEAM study, ten patients were excluded from randomization due to intolerance of the nociceptive test. Furthermore, multivariate analysis indicates that patients reporting adverse events were 1.33 times more likely to use acute migraine medication between 2–24 h compared to patients without adverse events. These findings highlight the importance of patient tolerability of the stimulation parameters in the compliance and success of migraine and migraine-associated symptom response.

In clinical practice, the e-TNS device has a “stabilization feature” (not utilized in this study) which allows patient to plateau or stabilize the stimulation intensity within the first 14 min if paresthesia becomes too intense^34^. This feature may help mitigate potential intolerance to stimulation of higher intensities, particularly with initial therapy. Therefore, it is crucial that patients are educated about the function and operation of this feature to optimize compliance and tolerability of the therapy^27^.

The practicality of a 2-h treatment duration may raise concern in terms of patient compliance. Overall, the compliance rates were higher for 60-min treatment duration compared to 2-h treatment. This finding may suggest a balance between optimal treatment time with neuromodulation therapy and desired pain relief from treatment. The rates of compliance and median duration of treatment in the TEAM study were similar to the prior open-label study, and this suggests that 2-h e-TNS treatments are feasible and practical alternatives to medicinal therapies in the real-world setting^16^.

## Limitations and future studies

Patients had one opportunity to correctly identify a qualifying migraine and apply and administer e-TNS treatment in an out of hospital setting. In attempts to limit potential selection bias, patients were required to distinguish between migraine and tension type headache as part of the study inclusion criteria. While an in-hospital setting may have improved accurate migraine attack identification, the current study was conducted outside of a hospital setting so that efficacy of e-TNS could be assessed in an environment where the device is commonly used (e.g., at home, without clinician supervision).

Ninety-four patients (17.5%) were unable to provide at least 60 min of therapy during a migraine. Self-administered treatments for a single migraine attack increased risk of incorrect device application, operation, and treatment disruption despite the 20-min training session. This is further complicated by possible interictal cognitive issues in patients with active migraine^31^. This observation highlights the importance of thorough device familiarization and instruction and underlies the importance of available, out-of-hospital patient education resources for proper e-TNS application and operation^27,35^. In addition to the device familiarization phase, future, self-application, neuromodulation clinical trials should provide patients thorough instructions for re-familiarization including online video tutorials and other training materials for the patient to review at home^27^.

The baseline number of headache years, average headache severity, usual preventative and acute anti-migraine treatments were not recorded and therefore their potential effect on the treatment outcomes could not be assessed. Although the at-home setting was permissive to early self-administered treatment, the specific timing of e-TNS intervention relative to migraine onset was not accurately assessed in this study. Migraine intervention timing may be a critical variable in symptom response, and further study is needed to elucidate this finding. Finally, longitudinal studies are needed to assess the consistency of the response of acute e-TNS treatment in migraine attacks.

## Conclusions

This was the first randomized, sham-controlled trial examining a 2-h e-TNS treatment for acute treatment of a migraine attack in an at-home scenario using a protocol design and outcomes for medications according to the Food and Drug Administration (FDA) and International Headache Society (IHS) guidance. The results suggests that 2-h e-TNS is an effective and safe, non-pharmacological, non-invasive treatment for acute treatment of a migraine attack with and without aura. E-TNS may be a clinically impactful alternative or complementary therapy in the stratified approach to acute management of migraine.

## Supplementary Information

Supplementary Table 1.

## Data Availability

The data that support the findings of this study are available from CEFALY technologies but restrictions apply to the availability of these data, which were used under license for the current study, and so are not publicly available. Data are however available from the authors upon reasonable request and with permission of CEFALY technologies.

## Abbreviations

ACME: Acute Migraine therapy with External Trigeminal Neurostimulation clinical trial
AE: Adverse events
e-TNS: External trigeminal nerve stimulation
ICHD-3: International Classification of Headache Disorders, Third edition
IQR: Interquartile ranges
ITT: Intention-to-treat
MBS: Migraine-associated bothersome symptom
MOH: Medication overuse headache
NSAIDS: Non-steroidal anti-inflammatory
SD: Standard deviations
PP: Per protocol
