# Supplementary material for: Phase 3 randomized, double-blind, sham-controlled Trial of e-TNS for the Acute treatment of Migraine (TEAM)
Source: Sci Rep. 2022 Mar 24;12:5110. doi: 10.1038/s41598-022-09071-6 (PMC8948251; doi:10.1038/s41598-022-09071-6)
Supplement: Supplementary file 1 — Supplementary Table 1. [file 41598_2022_9071_MOESM1_ESM.docx]

**Supplemental Table 1: Analgesic Medication use at 2-24 hours**

| **Acute Analgesic categories, # (%)** | **Intention to Treat** | | | **Per Protocol** | | |
| --- | --- | --- | --- | --- | --- | --- |
|  | **Verum**  **(*n*=259)** | **Sham**  **(*n*=279)** | **Total**  **(*N*=538)** | **Verum**  **(*n*=207)** | **Sham**  ***(n*=231)** | **Total**  **(*N*=438)** |
| NSAID | 14 (16.1) | 20 (17.9) | 34 (17.3) | 14 (20.3) | 19 (19.6) | 33 (19.9) |
| APAP | 8 (9.4) | 7 (6.3) | 15 (7.6) | 4 (5.8) | 7 (7.2) | 11 (6.6) |
| NSAID/Caffeine | 1 (1.2) | 1 (0.9) | 2 (1.0) | 1 (1.4) | 0 (0.0) | 1 (0.6) |
| APAP/Caffeine | 1 (1.2) | 0 (0.0) | 1 (0.5) | 1 (1.4) | 0 (0.0) | 1 (0.6) |
| APAP/ASA/Caffeine | 14 (16.5) | 25 (22.3) | 39 (19.8) | 8 (11.6) | 23 (23.7) | 31 (18.7) |
| Triptan | 38 (44.7) | 42 (37.5) | 80 (40.6) | 32 (46.4) | 32 (33.0) | 64 (38.6) |
| Triptan & NSAID | 5 (5.9) | 6 (5.4) | 11 (5.6) | 5 (7.2) | 6 (6.2) | 11 (6.6) |
| Triptan & Steroid | 0 (0.0) | 1 (0.9) | 1 (0.5) | 0 (0.0) | 1 (1.0) | 1 (0.6) |
| Anticonvulsant | 0 (0.0) | 2 (1.8) | 2 (1.0) | 0 (0.0) | 2 (2.1) | 2 (1.2) |
| Opioid | 1 (1.2) | 2 (1.8) | 3 (1.5) | 1 (1.4) | 2 (2.1) | 3 (1.8) |
| Butalbital/APAP/Caffeine | 0 (0.0) | 4 (3.6) | 4 (2.0) | 0 (0.0) | 4 (4.1) | 4 (2.4) |
| Antiemetic | 2 (2.4) | 1 (0.9) | 3 (1.5) | 2 (2.9) | 1 (1.0) | 3 (1.8) |
| Muscle Relaxant | 0 (0.0) | 1 (0.9) | 1 (0.5) | 0 (0.0) | 0 (0.0) | 0 (0.0) |
| Antihistamine | 1 (1.2) | 0 (0.0) | 1 (0.5) | 1 (1.4) | 0 (0.0) | 1 (0.6) |

Supplemental table 1: Summary of oral analgesic medication use at 2-24 hours following attempted treatment with e-TNS. Medications were self-reported by patients within 2 to 24 hours of e-TNS treatment and some patients used multiple (reference table 2). NSAID: Non-steroidal anti-inflammatory drug, APAP: acetaminophen, ASA: aspirin
